# Neutrophil-to-lymphocyte ratio as a predictor of poor outcomes of *Mycoplasma pneumoniae* pneumonia

**DOI:** 10.3389/fimmu.2023.1302702

**Published:** 2023-12-19

**Authors:** Dan Li, Haiyan Gu, Lei Chen, Ruxi Wu, Yazhou Jiang, Xia Huang, Deyu Zhao, Feng Liu

**Affiliations:** ^1^Department of Respiratory Medicine, Children’s Hospital of Nanjing Medical University, Nanjing, Jiangsu, China; ^2^Department of Pediatrics, Suqian Hospital Affiliated to Xuzhou Medical University, Suqian, Jiangsu, China

**Keywords:** neutrophil to lymphocyte ratio (NLR), *Mycoplasma pneumoniae* pneumonia (MPP), necrotizing pneumonia (NP), refractory *Mycoplasma pneumoniae* pneumonia (RMPP), outcomes

## Abstract

**Introduction:**

*Mycoplasma pneumoniae* pneumonia (MPP) may lead to various significant outcomes, such as necrotizing pneumonia(NP) and refractory MPP (RMPP). We investigated the potential of the peripheral blood neutrophil-to-lymphocyte ratio (NLR) to predict outcomes in patients with MPP.

**Methods and materials:**

This was a prospective study of patients with MPP who were admitted to our hospital from 2019 to 2021. Demographic and clinical data were collected from patient records and associated with the development of NP and RMPP and other outcome measures.

**Results:**

Of the 1,401 patients with MPP included in the study, 30 (2.1%) developed NP. The NLR was an independent predictor of NP (odds ratio 1.153, 95% confidence interval 1.022–1.300, *P*=0.021). The probability of NP was greater in patients with a high NLR (≥1.9) than in those with a low NLR (<1.9) (*P*<0.001). The NLR was also an independent predictor of RMPP (odds ratio 1.246, 95% confidence interval 1.102–1.408, *P*<0.005). Patients with a high NLR were more likely to develop NP and RMPP and require intensive care, and had longer total fever duration, longer hospital stays, and higher hospitalization expenses than those with a low NLR (all *P*<0.005).

**Discussion:**

The NLR can serve as a predictor of poor prognosis in patients with MPP. It can predict the occurrence of NP, RMPP, and other poor outcomes. The use of this indicator would allow the simple and rapid prediction of prognosis in the early stages of MPP, enabling the implementation of appropriate treatment strategies.

## 1 Introduction

*Mycoplasma pneumoniae* (MP) is a common pathogen that causes lower respiratory tract infections in children. MP pneumonia (MPP) accounts for approximately 32.4% of community-acquired pneumonia in Chinese children (1) and may be accompanied by various extrapulmonary complications. Incidences of severe MPP and refractory MPP (RMPP) have been increasingly reported in recent years (2, 3), which can lead to necrotizing pneumonia (NP) and a poor prognosis (4, 5). NP, which is diagnosed by radiography or computed tomography (CT), is characterized by multiple low-density, thin-walled cavities based on lung consolidation, sometimes containing a fluid level (6, 7). RMPP is defined as MPP with progressive exacerbation of clinical symptoms, persistent fever, and deterioration as determined by lung imaging after standard treatment with macrolide drugs for ≥7 d (8). Therefore, it is important to find early indicators to predict the severity of MPP, which can provide guidance for early clinical treatment.

Currently, known laboratory indicators that can predict the severity of MPP include peripheral blood neutrophil count, C-reactive protein (CRP), lactate dehydrogenase (LDH), interleukin-6, interleukin-17A, and D-dimer (9–12). Models predicting poor prognosis in MPP have also been established and evaluated (6, 13). However, it is well-known that neutrophil counts and CRP levels can be influenced by bacterial infection, and measuring other biomarkers requires invasive vein puncture. The neutrophil-to-lymphocyte ratio (NLR) in peripheral blood is a simple, rapid, and widely available indicator that has been reported to predict poor outcomes in various diseases, such as diabetes mellitus (14), cardiovascular disease (15), and cancer (16). In respiratory diseases, the NLR has been shown to be associated with poor prognosis in idiopathic pulmonary fibrosis (17), chronic obstructive pulmonary disease (14), and coronavirus disease-2019 (18). However, there is a lack of large cohort studies on the predictive value of the NLR for poor prognosis in children with MPP. In addition, there are few long-term follow-up studies on NP in the prognostic evaluation of MPP.

Therefore, we explored the relationship between the NLR and outcomes in children with MPP. We aimed to prospectively evaluate the association between the baseline NLR and prognosis in 1,401 children with MPP enrolled over a 3-year period. This may establish the NLR as a simple and rapid method for predicting poor prognosis in MPP, to guide early treatment and monitor disease progression.

## 2 Materials and methods

### 2.1 Participants

Patients with MPP who were admitted to the Respiratory Department of Children’s Hospital of Nanjing Medical University between January 1, 2019, and December 31, 2021, were included in this cohort study. We prospectively collected information on demographic data, clinical characteristics, laboratory tests, chest imaging findings, and clinical outcomes from electronic medical records. The study was approved by the Research Ethics Committee of our institution (approval number: 201812257-1; approval date: November 29, 2018). The requirement for informed consent was waived for this anonymized data.

### 2.2 Inclusion and exclusion criteria

Inclusion criteria: 1) aged ≥28 d and <18 y; 2) presence of respiratory symptoms or fever; 3) chest imaging confirmed pneumonia; and 4) MP-DNA positive nasopharyngeal secretions (≥1×10^3^ copies/mL) and positive serology. Exclusion criteria: 1) disease course ≥4 weeks prior to admission; 2) presence of other respiratory diseases, such as bronchial asthma, bronchopulmonary dysplasia, tuberculosis, primary ciliary dystrophy, cystic fibrosis, bronchial foreign body, pulmonary tumor, and non-infectious interstitial pulmonary disease; 3) presence of immunodeficiency, congenital heart disease, or heredity neurological disorders; 4) evidence of coinfection with other pathogens, as determined by sputum, blood, alveolar lavage fluid, pleural effusion culture, and virus testing; and 5) incomplete clinical data.

### 2.3 Data collection

Clinical information was prospectively collected from patient records. Age, sex, clinical symptoms and signs, extrapulmonary complications, preadmission fever duration, total fever duration, length of hospital stay, and hospitalization expenses were recorded. Allergy histories, including eczema, food and drug allergies, allergic rhinitis, allergic dermatitis, and allergic conjunctivitis, were recorded. Laboratory findings on admission, including white blood cell (WBC) counts, the NLR, CRP levels (BC-7500[NR]CS; Mindray, Shenzhen, China), alanine transaminase (ALT) levels, creatine kinase-MB levels, LDH levels (C502; Roche Diagnostics GmbH, Mannheim, Germany), blood coagulation function, and D-dimer levels (ACLTOP750; Werfen, Bedford, MA, USA), were recorded. Chest imaging findings were collected. Chest CT (Brilliance iCT; Philips Medical Systems, Cleveland, OH, USA) was performed when: 1) the clinical manifestations were inconsistent with the chest radiograph; 2) airway and lung malformations were suspected; 3) serious complications occurred; or 4) patients did not respond to treatment or had other diseases that needed to be excluded.

### 2.4 Clinical outcomes

Follow-up data were collected from electronic medical records, including primary (development of NP and RMPP) and secondary (total fever duration, length of hospital stay, hospitalization expenses, and referral to intensive care) outcomes.

The diagnosis of MP infection was based on MP-DNA positive nasopharyngeal secretions (≥1×10^3^ copies/mL) and positive serology (anti-MP IgM ≥1.0 COI). MP-DNA was detected by fluorogenic quantitative PCR (5700 Automatic PCR Analyzer; Perkin Elmer Inc., Wellesley, MA, USA). Anti-MP IgM was detected by chemiluminescent immunoassay (iFlash 3000; YHLO, Shenzhen, China).

### 2.5 Statistical analysis

SPSS software (version 25.0; IBM Corporation, Armonk, NY, USA) was used for statistical analysis. Normally distributed data are reported as mean ± SD; non-normally distributed data are reported as median (25^th^–75^th^ percentile). Categorical variables are expressed as frequencies and percentages. The groups were compared using a two-sample Student’s *t*-test (normally distributed data) or the Mann–Whitney *U* test (non-normally distributed data). Statistical significance was determined using the chi-square test and Fisher’s exact test. Logistic regression analysis was used to identify risk factors for MP-associated NP or RMPP. Multivariate analysis was performed using clinical characteristics with *P*<0.05 in the univariate analysis. Receiver operating characteristic (ROC) curves were plotted and the areas under the curve (AUCs) were calculated to compare the prognostic value of each variable. Survival curves were plotted using the Kaplan–Meier method and compared using the log-rank test. *P*<0.05 was considered statistically significant.

## 3 Results

### 3.1 Association between the NLR and NP

**Figure 1:**
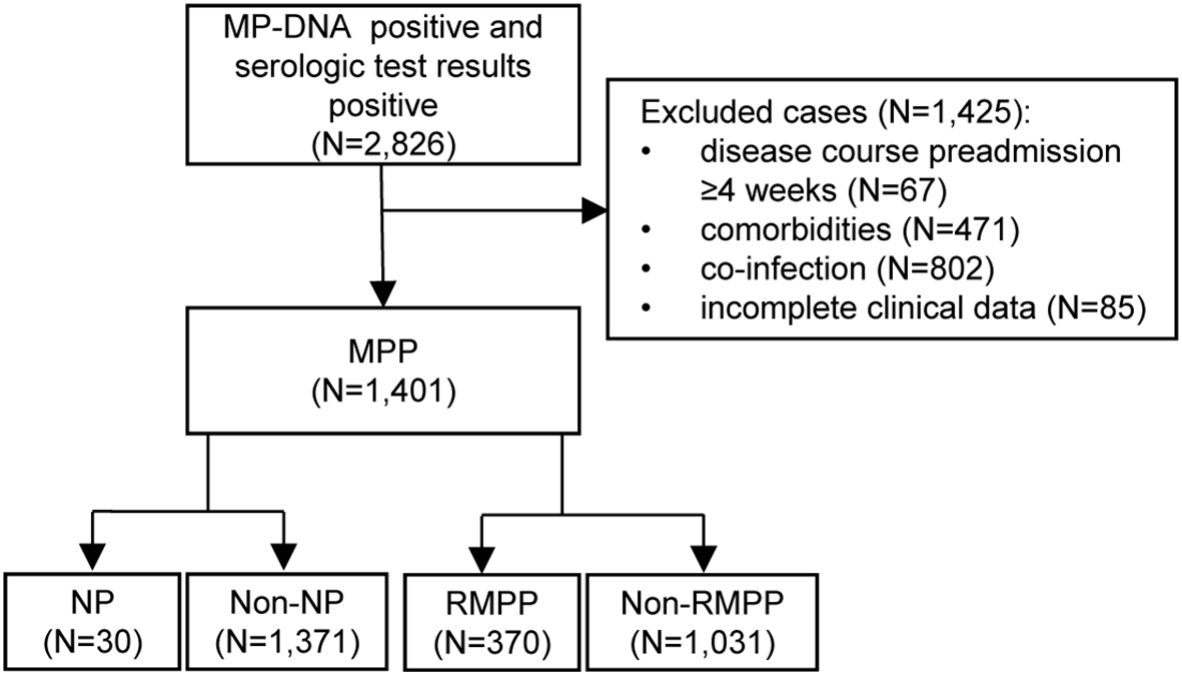
Flow chart of the study. MP, *Mycoplasma pneumoniae*; MPP, *Mycoplasma pneumoniae* pneumonia; NP, necrotizing pneumonia; RMPP, refractory *Mycoplasma pneumoniae* pneumonia.

In total, 9,661 patients with community-acquired pneumonia were considered for this study. Among them, 2,826 (29.3%) were MP-DNA positive and had positive serology. A total of 1,401 patients with MPP were included in the final analysis. All enrolled patients underwent chest X-ray or CT, which determined that 30 (2.1%) had developed NP. The study flowchart is presented in **Figure 1**.

The demographic and baseline clinical characteristics of the 1,401 patients are presented in **Table 1**. The patients were prospectively monitored for 60 d from the time of admission. There was no significant difference in the sex ratio between the NP and non-NP groups (*P*=0.191). Patients in the NP group were older than those in non-NP group (*P*=0.002). Patients with NP were more likely to have erythema, pleural effusion, and consolidation/atelectasis than those without NP (all *P*<0.05). Preadmission fever duration, WBC counts, the NLR, and CRP, LDH, ALT, and D-dimer levels were all significantly different between the NP and non-NP groups (all *P*<0.05).

**Table 1:** Demographic and baseline clinical characteristics of patients with MPP, with and without NP.

Characteristic	Total MPP (N=1,401)	NP (N=30)	Non-NP (N=1,371)	*P*-value
Sex, n (%)				
Male	817(58.3)	14(1.7)	803(98.3)	0.191
Female	584	16(2.7)	568(97.3)	
**Age, years**	4.8 (2.8–6.9)	6.1(5.3–7.1)	4.8(2.8–6.8)	**0.002**
Erythema, n (%)				
Yes	94(6.7)	6(6.4)	88(93.6)	**0.010**
No	1,307	24(1.8)	1,283(98.2)	
History of allergy, n (%)				
Yes	193(13.8)	3(1.6)	190(98.4)	0.735
No	1,208	27(2.2)	1,181(97.8)	
Moist rale, n (%)				
Yes	602(43.0)	5(0.8)	597(99.2)	**0.003**
No	799	25(3.1)	774(96.9)	
Wheeze, n (%)				
Yes	274(19.6)	3(1.1)	271(98.9)	0.182
No	1,127	27(2.4)	1,100(97.6)	
Pleural effusion, n (%)				
Yes	205(14.6)	24(11.7)	181(88.3)	**<0.001**
No	1,196	6(0.5)	1,190(99.5)	
Consolidation/atelectasis, n (%)				
Yes	566(40.4)	29(5.1)	537(94.9)	**<0.001**
No	835	1(0.1)	834(99.9)	
**Preadmission fever duration,** d	6.0 (4.0–8.0)	9.0(7.0–11.3)	6.0(4.0–8.0)	**<0.001**
**WBC,** ×10^9^/L	8.3 (6.6–10.7)	12.5(8.8–16.5)	8.2(6.6-10.6)	**<0.001**
**NLR**	1.9 (1.2–3.0)	6.0(3.8–9.3)	1.8(1.1- 2.9)	**<0.001**
Hemoglobin, g/L	125.0 (118.0–131.0)	121.0(109.5–130.3)	125.0(118.0-131.0)	0.054
Platelets, ×10^9^/L	269.5(208.0–342.3)	321.0(233.5–397.8)	269.0(208.0- 341.8)	0.203
**CRP,** mg/L	2.8(2.8–17.0)	54.0(16.0–92.0)	2.83(2.83–16.0)	**<0.001**
**LDH,** U/L	331.0 (287.0–408.5)	639.0(300.5–864.0)	331.0(287.0-405.0)	**<0.001**
**ALT,** U/L	14.0(10.0–21.0)	35.5(15.8–63.8)	14.0(10.0–20.0)	**<0.001**
CK-MB, U/L	23.0(18.0–28.0)	25.0(13.5–33.0)	23.0(18.0–28.0)	0.525
PT, s	12.3 (11.6–13.0)	12.2(11.2–13.1)	12.3(11.6-13.0)	0.793
APTT, s	31.7 (28.7–34.5)	29.1(26.8–35.2)	31.7(28.8- 34.4)	0.114
**D-dimer,** ng/mL	234.0 (143.0–539.0)	2872.0(1401.3–4398.5)	228.0(140.5-494.0)	**<0.001**
Fibrinogen, g/L	3.5 (2.9–3.9)	3.5(2.9–4.4)	3.5(2.9-3.9)	0.319

Data are presented as median (interquartile range) or n (%). ALT, alanine transaminase; APTT, activated partial thromboplastin time; CK-MB, creatine kinase-MB; CRP, C-reactive protein; LDH, lactate dehydrogenase; MPP, Mycoplasma pneumoniae pneumonia; NLR, neutrophil-to-lymphocyte ratio; NP, necrotizing pneumonia; PT, prothrombin time; WBC, white blood cells.

The bold values mean that the results were statistically significant(P<0.05).

Multivariate analysis showed that the NLR was associated with the development of NP after adjusting for other variables (odds ratio 1.153, 95% confidence interval 1.022–1.300, *P*=0.021) (**Table 2**). The NLR was, therefore, considered an independent risk factor for NP.

**Table 2:** Variables significantly associated with NP following multivariate analysis.

Variables	OR (95% CI)	*P*-value
Preadmission fever duration	1.137(1.007–1.284)	0.038
WBC	1.138(1.028-1.260)	0.013
NLR	1.153(1.022–1.300)	0.021
CRP	1.011(1.001–1.021)	0.034
LDH	1.003(1.001–1.005)	0.002
Pleural effusion		
Yes	3.823(1.294–11.288)	0.015
No		

CI, confidence interval; CRP, C-reactive protein; LDH, lactate dehydrogenase; NLR, neutrophil-to-lymphocyte ratio; OR, odds ratio; WBC, white blood cells.

**Figure 2:**
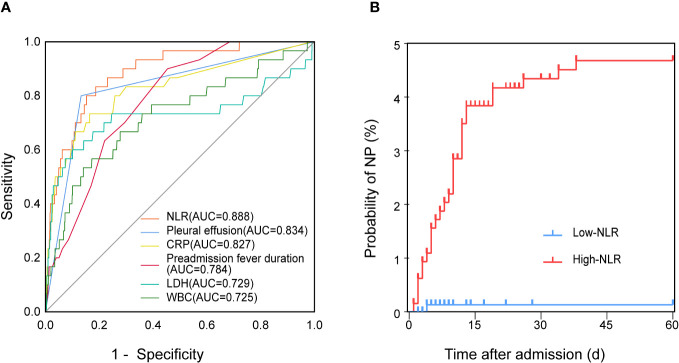
Relationship between the NLR and NP in patients with MPP. **(A)** ROC curves of the NLR, pleural effusion, CRP levels, preadmission fever duration, LDH levels, and WBC counts for predicting NP. **(B)** Kaplan–Meier curves of the incidence of NP in patients with a low (<1.9; N=708) and high (≥1.9; N=693) NLR at baseline. AUC, area under the curve; CRP, C-reactive protein; LDH, lactate dehydrogenase; MPP, *Mycoplasma pneumoniae* pneumonia; NLR, neutrophil-to-lymphocyte ratio; NP, necrotizing pneumonia; ROC, receiver operating characteristic; WBC, white blood cells.

ROC curves were generated for the variables found to be significant in the logistic regression analysis. The results showed that the NLR was a more effective predictor of NP than pleural effusion, CRP levels, preadmission fever duration, LDH levels, and WBC counts, with AUCs of 0.888, 0.834, 0.827, 0.784, 0.729, and 0.725, respectively (**Figure 2A**). The median NLR of the entire cohort (1.9) was used to divide patients into two groups: low (<1.9; N=708) and high (≥1.9; N=693) NLR groups. NP developed earlier, and the incidence of NP was significantly higher, in the high NLR group than in the low NLR group (*P*<0.001; **Figure 2B**).

### 3.2 Association between the NLR and RMPP

A total of 370 (26.4%) patients were diagnosed with RMPP. Patients in the RMPP group were older than those in the non-RMPP group (*P*<0.001). Patients with RMPP were more likely to have erythema, pleural effusion, and consolidation/atelectasis than those without RMPP (all *P*<0.005). Preadmission fever duration, WBC counts, the NLR, hemoglobin levels, platelet counts, prothrombin time, activated partial thromboplastin time, and CRP, LDH, ALT, and D-dimer levels were all significantly different between the RMPP and non-RMPP groups (all *P*<0.05; **Table 3**).

**Table 3:** Demographic and baseline clinical characteristics of patients with and without RMPP.

Characteristic	RMPP (N=370)	Non-RMPP (N=1,031)	*P*-value
Sex, n (%)			
Male	213(26.1)	604(73.9)	0.734
Female	157(26.9)	427(73.1)	
**Age, years**	6.1(4.3-7.6)	4.3(2.4-6.5)	**<0.001**
Erythema, n (%)			
Yes	40(42.6)	54(57.4)	**<0.001**
No	330(25.2)	977(74.8)	
History of allergy, n (%)			
Yes	34(17.6)	159(82.4)	**0.003**
No	336(27.8)	872(72.2)	
Moist rale, n (%)			
Yes	153(25.4)	449(74.6)	0.464
No	217(27.2)	582(72.8)	
Wheeze, n (%)			
Yes	37(13.5)	237(86.5)	**<0.001**
No	333(29.5)	794(70.5)	
Pleural effusion, n (%)			
Yes	156(76.1)	49(23.9)	**<0.001**
No	214(17.9)	982(82.1)	
Consolidation/atelectasis, n (%)			
Yes	312(55.1)	254(44.9)	**<0.001**
No	58(6.9)	777(93.1)	
**Preadmission fever duration,** d	8.5(6.8-10.0)	5.0(3.0-7.0)	**<0.001**
**WBC**, ×10^9^/L	9.1(6.8-12.4)	8.0(6.6-10.2)	**<0.001**
**NLR**	3.0(1.8-4.8)	1.6(1.0-2.5)	**<0.001**
**Hemoglobin**, g/L	123.0(115.0-130.0)	125.0(119.0-131.0)	**<0.001**
**Platelets,** ×10^9^/L	292.0(221.5-363.0)	262.0(205.0-331.3)	**<0.001**
**CRP,** mg/L	13.0(2.8-34.3)	2.8(2.8-13.0)	**<0.001**
**LDH,** U/L	405.0(316.8-570.5)	320.0(280.0-374.0)	**<0.001**
**ALT,** U/L	18.0(12.0-35.3)	13.0(10.0-17.0)	**<0.001**
CK-MB, U/L	23.0(17.0-28.0)	23.0(18.0-29.0)	0.081
**PT,** s	12.4(11.6-13.4)	12.2(11.6-12.9)	**0.016**
**APTT,** s	30.1(27.1-33.2)	32.2(29.5-34.8)	**<0.001**
**D-dimer,** ng/mL	677.0(284.0-1901.0)	186.5(124.0-288.8)	**<0.001**
Fibrinogen, g/L	3.5(2.9-4.0)	3.5(3.0-3.9)	0.320

Data are presented as median (interquartile range) or n (%). ALT, alanine transaminase; APTT, activated partial thromboplastin time; CK-MB, creatine kinase-MB; CRP, C-reactive protein; LDH, lactate dehydrogenase; NLR, neutrophil-to-lymphocyte ratio; PT, prothrombin time; RMPP, refractory Mycoplasma pneumoniae pneumonia; WBC, white blood cells.

The bold values mean that the results were statistically significant(P<0.05).

Multivariate analysis showed that the NLR was associated with the development of RMPP after adjusting for other variables (odds ratio 1.246, 95% confidence interval 1.102–1.408, *P*<0.001) (**Table 4**). The NLR was, therefore, considered an independent risk factor for RMPP.

**Table 4:** Variables significantly associated with RMPP following multivariate analysis.

Variables	OR (95% CI)	*P*-value
Preadmission fever duration	1.159(1.100-1.220)	<0.001
WBC	1.092(1.024-1.164)	0.007
NLR	1.246(1.102-1.408)	<0.001
LDH	1.003(1.001-1.004)	<0.001
ALT	1.009(1.001-1.017)	0.025
Consolidation/atelectasis		
Yes	6.216(4.223-9.149)	<0.001
No		
Pleural effusion		
Yes	2.752(1.703-4.449)	<0.001
No		

ALT, alanine transaminase; CI, confidence interval; CRP, C-reactive protein; LDH, lactate dehydrogenase; NLR, neutrophil-to-lymphocyte ratio; OR, odds ratio; WBC, white blood cells.

**Figure 3:**
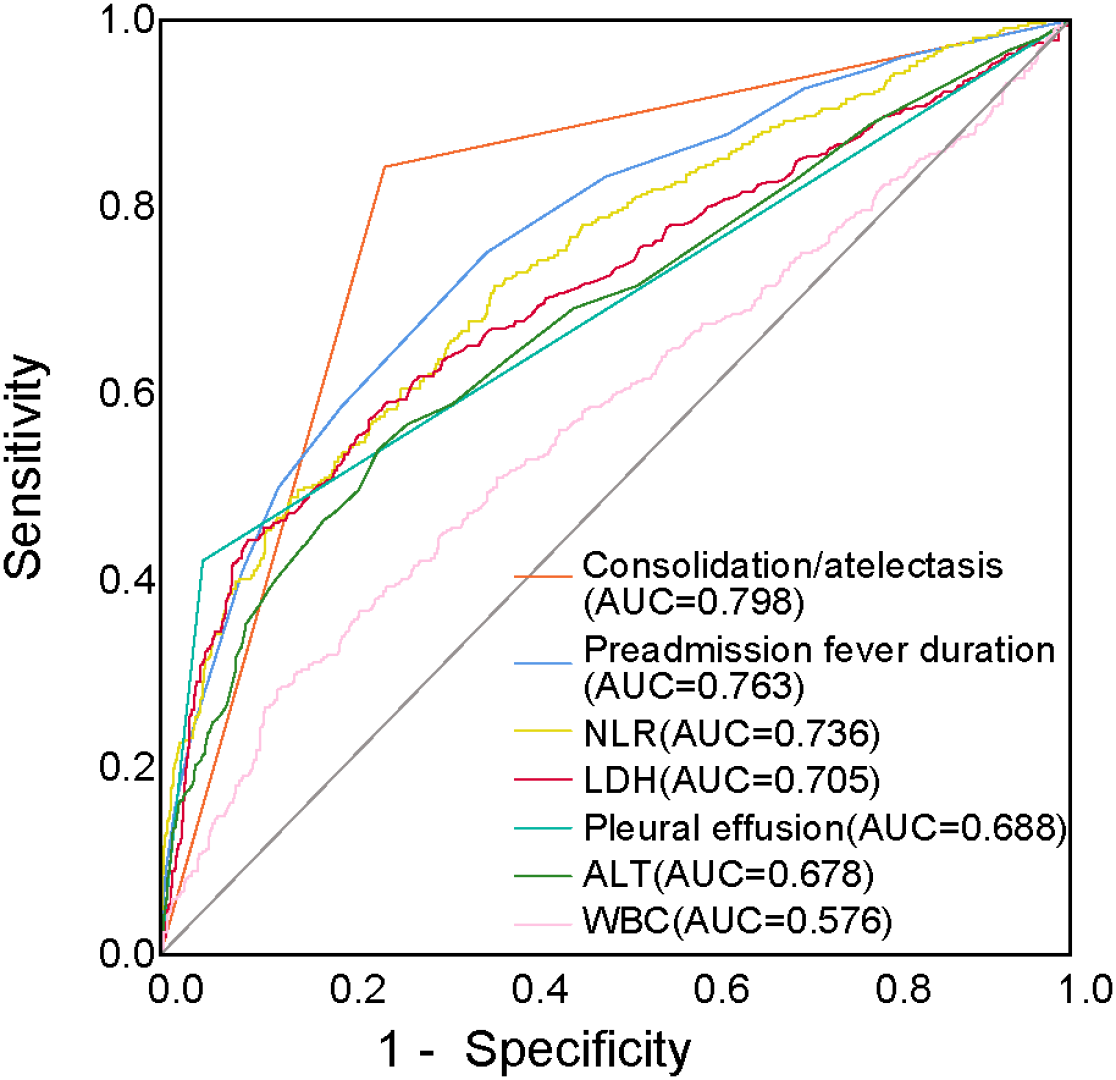
ROC curves of the NLR, consolidation/atelectasis, preadmission fever duration, LDH levels, pleural effusion, ALT levels, and WBC counts for predicting RMPP. ALT, alanine transaminase; AUC, area under the curve; LDH, lactate dehydrogenase; NLR, neutrophil-to-lymphocyte ratio; RMPP, refractory *Mycoplasma pneumoniae* pneumonia; ROC, receiver operating characteristic; WBC, white blood cells.

ROC curves were generated for the variables found to be significant in the logistic regression analysis. The results showed that the NLR effectively predicted RMPP, with an AUC of 0.736. The AUCs for consolidation/atelectasis, preadmission fever duration, LDH levels, pleural effusion, ALT levels, and WBC counts were 0.798, 0.763, 0.705, 0.688, 0.678, and 0.576, respectively (**Figure 3**).

### 3.3 Association between the NLR and other outcomes

The incidences of NP and RMPP, intensive care unit (ICU) admission, total fever duration, length of hospital stay, and hospitalization expenses were compared between the high (≥1.9; N=693) and low (<1.9; N=708) NLR groups. The high NLR group had higher incidences of NP and RMPP and ICU admission, longer total fever duration and hospital stay, and higher hospitalization expenses than those in the low NLR group (all *P*<0.005; **Table 5**).

**Table 5:** Outcomes of patients with high and low NLRs.

Characteristic	High NLR (N=693)	Low NLR (N=708)	*P*-value
NP, n (%)	29 (4.2)	1 (0.1)	<0.001
RMPP, n (%)	272 (39.2)	98 (13.8)	<0.001
ICU, n (%)	15 (2.2)	3 (0.4)	0.004
Total fever duration, d	8.0 (5.0-10.0)	6.0 (3.0-7.0)	<0.001
Length of hospital stay, d	8.0 (6.0-10.0)	7.0 (6.0-8.0)	<0.001
Hospitalization expenses, CNY	8483.5 (6593.6-12634.6)	6698.1 (5456.3-8830.4)	<0.001

Data are presented as median (interquartile range) or n (%). CNY, Chinese Yuan; ICU, intensive care unit; RMPP, refractory Mycoplasma pneumoniae pneumonia; NLR, neutrophil-to-lymphocyte ratio; NP, necrotizing pneumonia.

## 4 Discussion

The present study prospectively investigated the association between the NLR in peripheral blood and poor outcomes in patients with MPP who were admitted to our department from 2019 to 2021. We set the occurrence of NP as a major adverse outcome. Of the 1,401 patients with MPP, 30 (2.1%) developed NP. This is consistent with the proportion reported by Luo and Wang (6), in a retrospective study of 3,872 children with MPP, 84 (2.2%) of whom developed NP. NP is a rare complication of community-acquired pneumonia, and is most commonly associated with *Streptococcus pneumoniae* and *Staphylococcus aureus* (19). However, since Oermann et al. (20) first reported NP resulting from MP infection in 1997, an increasing number of cases have been reported. NP does not usually occur in the early stages of MPP, and diagnosis requires monitoring by chest CT. NP causes considerable lung damage in patients with MPP and recovery is slow. Therefore, early and easy-to-measure prognostic biomarkers are necessary for a better understanding of the clinical course and risk stratification of patients with MPP, which would also permit the early use of glucocorticoids, to improve the poor prognosis.

In our study, patients with erythema, pleural effusion, and consolidation/atelectasis had higher incidences of NP and RMPP than those without these complications, which are associated with the systemic inflammatory response to MP infection (21). We also found that preadmission fever duration, WBC counts, the NLR, and CRP, LDH, ALT, and D-dimer levels were higher in patients with NP or RMPP than those without NP or RMPP. High WBC counts, NLR, and CRP, LDH, and D-dimer levels are indicators of inflammation that reflect a stronger systemic inflammatory response in patients with NP or RMPP than in those without NP or RMPP. These findings indicate that excessive inflammatory and immune responses by the host play important roles in the pathogenesis of NP and RMPP. Our study showed that patients with NP or RMPP were older than those without NP or RMPP.

Our study indicates that the NLR is an independent risk factor for NP and RMPP caused by MP. ROC analysis showed that the NLR predicted the incidence of NP with greater accuracy than pleural effusion, preadmission fever duration, WBC counts, and CRP and LDH levels. The NLR and neutrophil counts are recognized parameters of MPP disease progression. A retrospective observational study (10) reported that a WBC count >12.3×10^9^/L, a neutrophil count >73.9%, and D-dimer levels >1,367.5 ng/mL were risk factors for pulmonary necrosis caused by MP. Another retrospective study (9) concluded that an NLR >3.92 may have important predictive value for RMPP in children over 6 years. However, few studies have focused on the predictive value of the NLR in NP and other adverse outcomes of MPP in children. Our study showed that NP developed earlier, and the incidence of NP was significantly higher, in the high NLR group (≥1.9) than in the low NLR (<1.9) group. Patients with a high NLR were more likely to develop NP or RMPP and require ICU admission than those with a low NLR. They also had a longer total fever duration and hospital stay and higher hospitalization expenses.

The NLR is an inexpensive, widely available, and easily measured biomarker. Neutrophils and lymphocytes are essential components of the immune system. Neutrophils, as part of the first line of defense against infections, play a crucial role in the immune response to MP (22), and their numbers in peripheral blood (23), bronchoalveolar lavage fluid (24), and lung tissue (25) increase after MP infection. Neutrophils interact with other cells, including endothelial cells and platelets, to form a link between inflammation and thrombosis (26); overactivation can lead to tissue damage (27). However, excessive inflammation may induce the apoptosis of lymphocytes, resulting in a decrease in their numbers (28).

Our results indicate that the NLR at admission can predict the prognosis of MPP, providing early treatment guidance. As an inexpensive, easily accessible indicator that is independently associated with NP, RMPP, and other adverse outcomes of MPP, the NLR is a promising prognostic biomarker. It is also easily obtained in primary hospitals prior to CT, allowing early identification and referral of critically ill patients. Future large, prospective, multicenter, cohort studies are needed to confirm the predictive value of the NLR for MPP.

## Data availability statement

The raw data supporting the conclusions of this article will be made available by the authors, without undue reservation.

## Ethics statement

The studies involving humans were approved by Ethical approval was granted by the research ethics committee of Children’s Hospital of Nanjing Medical University (approval number: 201812257-1). The studies were conducted in accordance with the local legislation and institutional requirements. Written informed consent for participation was not required from the participants or the participants’ legal guardians/next of kin in accordance with the national legislation and institutional requirements.

## Author contributions

DL: Data curation, Formal analysis, Investigation, Software, Writing – original draft, Writing – review & editing. HG: Funding acquisition, Writing – review & editing, Investigation, Data curation, Project administration. LC: Investigation, Writing – review & editing. RW: Investigation, Writing – review & editing. YJ: Formal analysis, Writing – review & editing. XH: Software, Writing – review & editing. DZ: Conceptualization, Methodology, Project administration, Supervision, Writing – review & editing. FL: Funding acquisition, Supervision, Writing – review & editing, Formal analysis, Investigation, Validation.
